# Identifying High-Risk Pre-Term Pregnancies Using the Fetal Heart Rate and Machine Learning

**DOI:** 10.3390/bioengineering13020203

**Published:** 2026-02-11

**Authors:** Gabriel Davis Jones, William R. Cooke, Manu Vatish

**Affiliations:** 1 Oxford Digital Health Labs, Nuffield Department of Women’s & Reproductive Health, University of Oxford, Oxford OX3 9DU, UK; 2 Nuffield Department of Women’s & Reproductive Health, University of Oxford, Oxford OX3 9DU, UK

**Keywords:** fetal heart rate monitoring, cardiotocography, pre-term birth, machine learning, risk stratification, antepartum surveillance, perinatal outcomes

## Abstract

Fetal heart rate (FHR) monitoring is ubiquitous in antenatal care, yet human visual interpretation poorly predicts adverse pregnancy outcomes. Meanwhile, preterm gestations carry a high burden of stillbirth and severe fetal compromise, where earlier identification of high-risk pregnancies may justify iatrogenic preterm delivery to prevent avoidable fetal death. We analyzed 4867 antepartum FHR recordings from pre-term pregnancies meeting at least one of ten adverse outcome criteria alongside 4014 term uncomplicated controls. Seven clinically validated FHR features were extracted from each trace, and six machine-learning classifiers were trained on 80% of the data (7105 samples) using k-fold cross-validation; the remaining 20% (1776 samples) formed an internal validation cohort. The random forest demonstrated the best performance, achieving an area under the receiver-operating characteristic curve (AUC) of 0.88 (95% confidence interval [CI] 0.87–0.88) during training and 0.88 (95% CI 0.86–0.90) on validation, with good calibration (Brier score 0.14). Median AUC across individual adverse outcomes was 0.85 (interquartile range [IQR] 0.81–0.89) and exceeded 0.80 at all gestational ages assessed; sensitivity and specificity at the Youden threshold were 76.2% and 87.5%, respectively. Decision-curve analysis demonstrated net benefit across a range of clinically relevant probability thresholds. These findings indicate that data-driven interpretation of antepartum FHR can stratify risk in pre-term pregnancies with high accuracy and may support earlier, evidence-based clinical decision-making, particularly in resource-limited settings where specialist expertise is limited.

## 1. Introduction

Fetal heart rate (FHR) monitoring is one of the most widely used antepartum obstetric investigations, applied in more than 85% of pregnancies worldwide [1]. A non-invasive ultrasound transducer placed on the maternal abdomen records continuous FHR signals—the “non-stress test” or “cardiotocography”—providing real-time assessment of fetal autonomic nervous system activity and overall physiological status [2,3].

Characteristic FHR patterns act as proxy measures of fetal neurological integrity and overall wellbeing [4,5]. During the third trimester, these signals guide clinicians in identifying fetuses at risk of adverse pregnancy outcomes and in deciding whether enhanced surveillance or early intervention is required [6,7,8]. Despite six decades of clinical use, visual interpretation of antepartum FHR traces remains unreliable. Expert observers misclassify 35–92% of patterns [9,10,11], and inter- and intra-observer agreement can be as low as 29% [12,13,14,15,16,17]. High false-positive rates (up to 60%) are associated with unnecessary interventions, increased maternal and neonatal morbidity, and a risk of potentially avoidable adverse outcomes—including fetal death—as well as substantial medicolegal liability [18,19,20,21].

Standardisation initiatives have not resolved issues of performance, reproducibility, or consensus [21,22,23,24,25]. Perinatal mortality disproportionately affects low- and middle-income countries, which account for 98% of deaths [26]. Among available fetal assessment technologies, FHR monitoring is relatively inexpensive and requires minimal technical training [26,27]. However, the specialised training required to interpret complex traces represents a major barrier to wider and more equitable adoption of this technology.

Recent advances in machine learning and the availability of large clinical datasets have improved early detection of pregnancy disorders [28,29,30,31], and algorithms now outperform clinicians in several diagnostic domains [32,33]. Although intrapartum FHR analysis using machine learning has shown promise [34,35,36], antepartum, pre-term (≤37 weeks) monitoring remains understudied, despite its clinical importance [37]. Identifying fetuses at genuine risk of adverse outcome at preterm gestations is notoriously difficult, contributing both to persistently high rates of stillbirth and to avoidable iatrogenic preterm birth arising from diagnostic uncertainty. Complications of pre-term birth represent the leading global cause of death in children ≤ 5 years of age, responsible for approximately one million deaths each year [38,39].

To date, most computational and machine learning approaches applied to cardiotocography have focused on the intrapartum period, where FHR signals are analyzed during labor to predict acute fetal compromise or to replicate expert-defined CTG classifications [40,41,42]. These studies have demonstrated automated analysis can match or exceed human performance in identifying intrapartum fetal distress; however, they are typically limited to term pregnancies, short recording windows, and outcomes that reflect expert interpretation rather than objective neonatal or perinatal endpoints. Many machine learning studies also rely on small, highly curated datasets, such as the publicly available UCI cardiotocography dataset, which restricts translation to routine clinical practice [43].

In contrast, there is a notable paucity of published work applying machine learning methods to antepartum cardiotocography, particularly in the pre-term setting. Antepartum recordings differ fundamentally from intrapartum traces in both underlying physiology and clinical context, and findings derived from labor cannot be assumed to generalise to earlier antepartum gestations. To our knowledge, no large-scale study has systematically evaluated machine learning models trained on clinically validated FHR features to predict objectively defined adverse outcomes in pre-term pregnancies using antepartum CTG. This lack of evidence represents a critical gap, given that antepartum surveillance is the primary opportunity for early identification and intervention in high-risk pre-term pregnancies.

Here, we assemble a large, real-world cohort of high-risk pre-term pregnancies to evaluate whether machine learning models trained on clinically validated fetal heart rate patterns can identify fetuses at heightened risk of adverse outcomes. We focus on pregnancies undergoing antepartum cardiotocography for established clinical indications, rather than proposing universal screening. Predictive performance is assessed across gestational ages relevant to pre-term surveillance, and potential clinical utility is evaluated using decision-curve analysis alongside standard measures of discrimination and calibration. Through this approach, we aim to determine whether data-driven interpretation of antepartum pre-term FHR can improve risk stratification within existing models of selective antenatal surveillance and support more informed clinical decision-making.

## 2. Methods

### 2.1. Data Processing, Study Group Identification and Extraction of Fetal Heart Rate Features

We extracted raw digital antepartum FHR traces from the Oxford University Hospitals maternity database at the John Radcliffe Hospital (Oxford, UK) between 30 November 1990 and 31 December 2021. This study was approved by the Ethics Committee in the Joint Research Office, Research and Development Department, Oxford University Hospitals NHS Trust (approval number: 25/HRA/1966). Traces were acquired from singleton pregnancies between 27^+0^ and 36^+6^ gestational weeks for which complete maternal and neonatal associated clinical outcome data were available. We defined two study cohorts: a normal pregnancy outcome (NPO) cohort delivered at term and a high-risk adverse pregnancy outcome (APO) cohort of pre-term delivery pregnancies as previously described [8]. Strict inclusion and exclusion criteria were used to obtain a normal cohort (Supplementary Table S1) to minimise confounding. Eligible records were from pregnant women aged between 18–39 years with a BMI ≤ 30 kg/m^2^, normal pregnancy biomarker and ultrasound scan findings, term delivery (37^+0^–41^+0^ weeks), birthweights between 25th–75th centiles, normal Apgar scores (≥4 at 1 min; ≥7 at 5 min), and no requirement for neonatal resuscitation or special care admission following delivery. For pregnancies with more than one FHR trace available in a gestational week, only the first trace was included to minimise potential bias arising from repeat recordings prompted by findings on the initial trace.

The high-risk preterm APO comprised pregnancies in which the baby met at least one predefined adverse outcome criterion at delivery. These included biochemical evidence of acidaemia, antepartum/intrapartum stillbirth, perinatal asphyxia, a birthweight ≤ 3 rd centile for gestational age [44], an extended special care admission ≥ 7 days, hypoxaemic ischemic encephalopathy, low Apgar scores, neonatal sepsis, perinatal infections, or respiratory conditions. The definition of acidaemia is an arterial pH < 7.13 and arterial base deficit > 10.0 for babies delivered via caesarean section without labor or arterial pH < 7.05 and arterial base deficit > 14.0 for babies who experienced labor irrespective of mode of delivery in accordance with hospital guidelines where the data were acquired. Low Apgar scores were defined as <4 at 1 min and <7 at 5 min [45]. Asphyxia was defined as low Apgar scores in conjunction with acidaemia. Hypoxaemic ischemic encephalopathy and neonatal sepsis were diagnosed by consultant neonatologists registered on the UK General Medical Council Specialist Register (equivalent to board-certified). Diagnoses were obtained either directly from clinical records or using Phecodes (Phecode version 1.2; perinatal infection: 657, respiratory conditions: 656.2) [46].

We excluded records from babies delivered with incomplete or inadequate outcome information to avoid potential confounding. Because antepartum FHR monitoring informs clinical decision making and may precipitate early delivery, inclusion of preterm traces in the absence of independently verifiable adverse outcomes could introduce indication bias. To further mitigate temporal misclassification, we also excluded traces from the high-risk preterm adverse outcome cohort that were acquired more than 7 days prior to delivery. Antepartum cardiotocography is performed for myriad indications throughout pregnancy. It is therefore unreliable to assume traces acquired throughout pregnancy are for a consistent indication. Incorporating traces acquired substantially earlier than the outcome was identified without clinical evidence would assume all traces acquired for that pregnancy were performed while the same pathology was present in the fetus. Constraining the time window for adverse outcome traces to within 7 days prior to delivery assists in avoiding this assumption.

We processed the raw antepartum FHR signals with an established automated feature identification algorithm to extract seven features [8,34]: basal FHR, accelerations, decelerations, most lost beats (MLB), short-term variation (STV), time spent in an episode of high variation, and time spent in an episode of low variation (minutes). To ensure consistency across recordings, features were not extracted beyond 60 min of the trace.

These features and their extraction methods have previously been described and clinically validated in the literature [47]. We provide here a brief description of these features. The first procedure in FHR feature extraction is fitting a baseline (the average FHR excluding any major deviations) to the signal. This serves as the reference point for the trace, facilitating the identification of other features. Accelerations and decelerations are transient deviations above or below this baseline. An acceleration is defined as a temporary increase in the FHR of at least 10 bpm above the baseline lasting longer than 15 s. A deceleration is a decrease of at least 20 bpm lasting longer than 30 s or at least 10 bpm lasting longer than 60 s. “Lost beats” is the product of the duration of the deceleration and the magnitude of the deviation from the baseline of deceleration. “Most lost beats” is the largest observed loss of beats due to a deceleration in an FHR trace. STV is the mean absolute difference in time intervals between successive heart rate pulses. Episodes of high and low variation are defined as episodes in which the variability of the FHR trace is consistently above (high variation) or below (low variation) pre-determined thresholds of variation.

Fetal movements were not included in our analysis, as they are a subjective measurement and inconsistently recorded in a clinical setting. We then examined each trace for physiologically implausible or poor quality outliers, excluding any trace that demonstrated >30% signal loss, basal FHR < 100 or >180 bpm, >1 acceleration per minute, >125 most lost beats, or an STV < 2 or >30 ms based on established clinical thresholds. Feature transformation was performed to place all variables on comparable numerical scales prior to model training. Features with approximately symmetric distributions were standardised using z-score normalisation, calculated as the feature value minus the mean divided by the standard deviation. Features with skewed distributions were transformed using min-max normalisation to rescale values to the interval [0, 1]. No dimensionality reduction was performed, and all seven transformed FHR features were retained as model inputs. Normalisation parameters were derived from the training dataset and applied unchanged to the validation dataset.

We performed propensity score matching, matching for gestational age at FHR trace acquisition, fetal sex, and trace duration. The K-nearest neighbors algorithm was used to sample without replacement, matching each identified case of a preterm adverse outcome to a normal outcome pregnancy where available. The data were then randomly spliced 80:20% into a model training dataset and internal validation dataset, balanced for outcome, gestational age, trace duration, and fetal sex.

**Figure 1:**
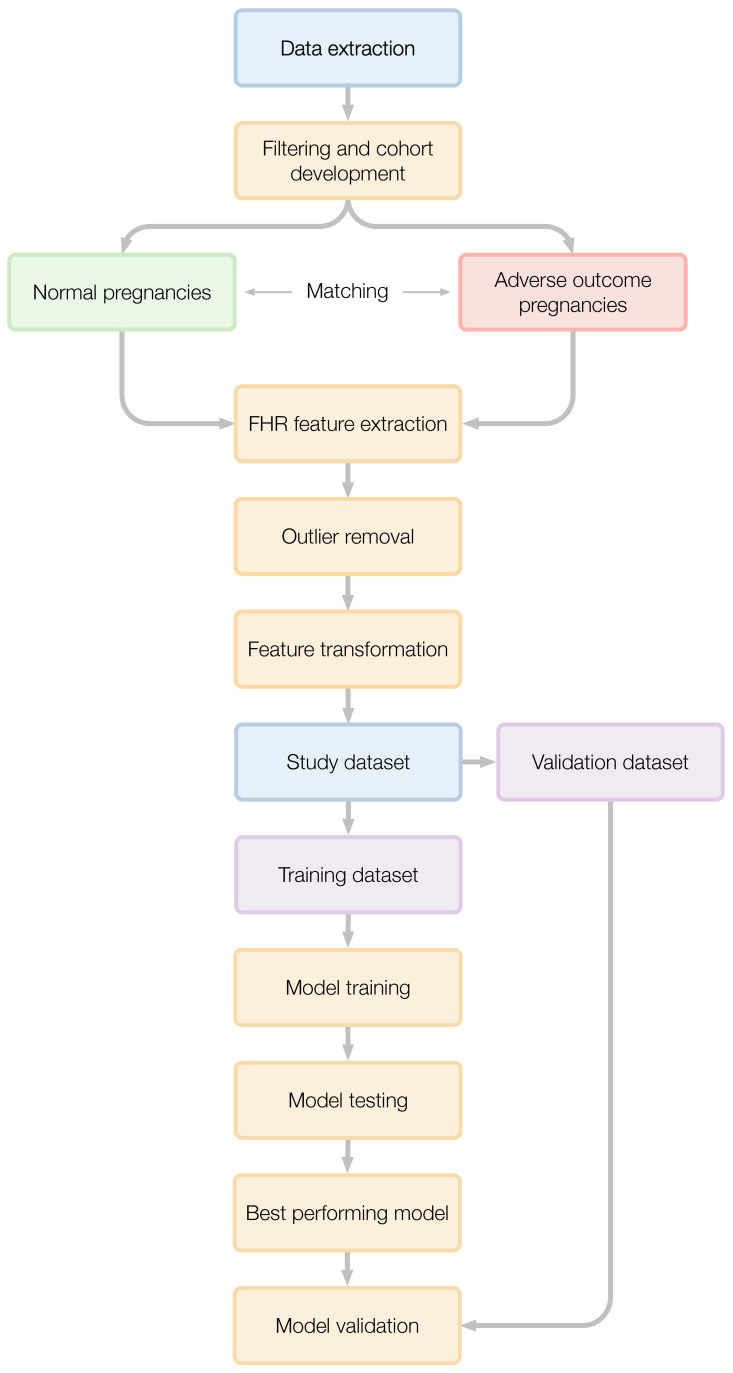
Data flow for the development of a study dataset and predictive model to identify pre-term adverse outcome pregnancies using the antepartum FHR.

Figure 1 summarises the full data processing and modeling pipeline. Raw antepartum FHR recordings were extracted and filtered to define normal pregnancy outcome and preterm adverse outcome cohorts. Following propensity score matching, clinically validated FHR features were extracted from each trace, outliers were removed, and features were transformed to ensure comparability across different scales. The resulting dataset was then split into training and internal validation sets for model development, testing, and final validation.

After preprocessing and transformation, each sample consisted of a seven-dimensional feature vector corresponding to the extracted FHR features from a single antepartum trace. Each sample was associated with a binary label indicating either a normal pregnancy outcome or a preterm adverse outcome. Following matching and quality control, the final dataset comprised 8881 samples. These were randomly partitioned into a training dataset containing 7105 samples (80%) and an internal validation dataset containing 1776 samples (20%), with class balance preserved across both datasets.

### 2.2. Development of Machine Learning Models

We trained six machine learning algorithms on the model training dataset to predict whether a trace belonged to the normal (NPO) or preterm adverse outcome (APO) pregnancy. The algorithms were a decision tree (DT), Gaussian naïve Bayes (GNB), logistic regression (LR), random forest (RF), support vector machine (SVM), and XGBoost (XGB). These algorithms were selected because they are widely used in clinical predictive modeling, encompass a range of linear and non-linear decision boundaries, and vary in complexity and interpretability [48]. In perinatal medicine, preterm adverse outcomes frequently exhibit concomitance; for example, low Apgar scores are often associated with conditions such as hypoxaemic ischemic encephalopathy and neonatal sepsis. Consequently, we opted against the development of a multiclass predictive model designed to identify each individual adverse outcome.

Each algorithm was trained using the transformed values of the seven FHR features. 10-fold cross-validation was used, with each fold balanced for outcome, gestational age, trace duration, and fetal sex. The optimum hyperparameters for each algorithm were identified using Bayesian optimisation. The average receiver-operator characteristic area under the curve (AUC) for each model was then used to evaluate the model’s overall performance. We ranked each model by AUC and compared the median AUC between each model with a Kruskal-Wallis one-way ANOVA and performed a pair-wise Mann–Whitney U test with a significance threshold of 0.01. The best-performing model was then selected for subsequent evaluation on the internal validation dataset.

The selected model was then evaluated on the internal validation dataset using the area under the receiver operator characteristic curve (AUC), sensitivity (of everyone classified as belonging to the preterm adverse outcome group, how many did the model correctly identify), specificity (how many normal FHR traces were correctly identified as such), F1 score (the harmonic mean of the proportion of true positives among the identified positives and sensitivity) and Cohen’s Kappa (the level of agreement between the predictive model and the known outcome). We determined that for a predictive model to demonstrate significant potential benefit, the average AUC must exceed 0.70 (in keeping with similar studies from the intrapartum period) [36,49]. An AUC of <0.6 would suggest poor discrimination, while 0.6–0.69 would be fair, 0.70–0.79 would be good, and >0.8 would be excellent, in keeping with similar publications [50]. We evaluated the AUC across all gestational ages and for each gestational age between 27^+0^ and 36^+0^ weeks.

Decision curve analysis was performed to compare the net benefit of the model against “treat-all” and “treat-none” strategies. In this framework, treat-all corresponds to managing all pregnancies as high-risk, while treat-none represents no additional intervention. Net benefit was evaluated across probability thresholds ranging from 0.01 to 0.99 to assess whether the model improved identification of high-risk pregnancies while reducing unnecessary interventions. Model calibration was assessed using the Brier score.

### 2.3. Statistical Analysis

We adhere to TRIPOD guidelines for reporting [51] (see Supplementary Data). Discrete variables are presented as numbers (with interquartile ranges) and percentages, while continuous variables are listed as mean and 95% confidence intervals (95% CI). Categorical variables were compared using the chi-square test, while continuous variables were compared using the Mann-Whitney U test with a significant threshold of 0.05. Predictive models were compared using an ANOVA and pair-wise Mann–Whitney U test with a significance threshold of 0.01. *p*-values were estimated for each feature’s association with a high-risk pregnancy using the Mann–Whitney U test and a significance threshold of 0.05. Confidence intervals were calculated using the bootstrap method. Effect sizes were analyzed using Cohen’s D for parametric and rank-biserial correlation for non-parametric variables. Analysis was performed using Python (version 3.9.17) with the Pandas (version 1.5.3), NumPy (version 1.23.5), Matplotlib (version 3.7.1), and SciPy (version 1.10.1) packages.

## 3. Results

The study population comprised 8881 antepartum FHR traces, including 4014 (45.2%) normal pregnancy outcome (NPO) traces and 4867 (54.8%) high-risk preterm adverse pregnancy outcome (APO) traces (Table 1). The median maternal age was 30 years (interquartile range [IQR] 25–34), median parity was 1 (IQR 0–1), and median BMI was 23.5 kg/m^2^ (IQR 21.3–26.2). Of the included FHR traces, 4335 (48.8%) were from male fetuses and 4546 (51.2%) were from female fetuses. The distribution of outcomes did not differ significantly across gestational ages (*p* = 0.17). All seven FHR features differed significantly between the NPO and APO groups (Table 2). Median values for accelerations, episodes of high variation, and short-term variability were higher in the NPO group (all *p* <0.001), while basal heart rate, decelerations, episodes of low variation, and most lost beats were higher in the APO group (all *p* <0.001).

**Figure 2:**
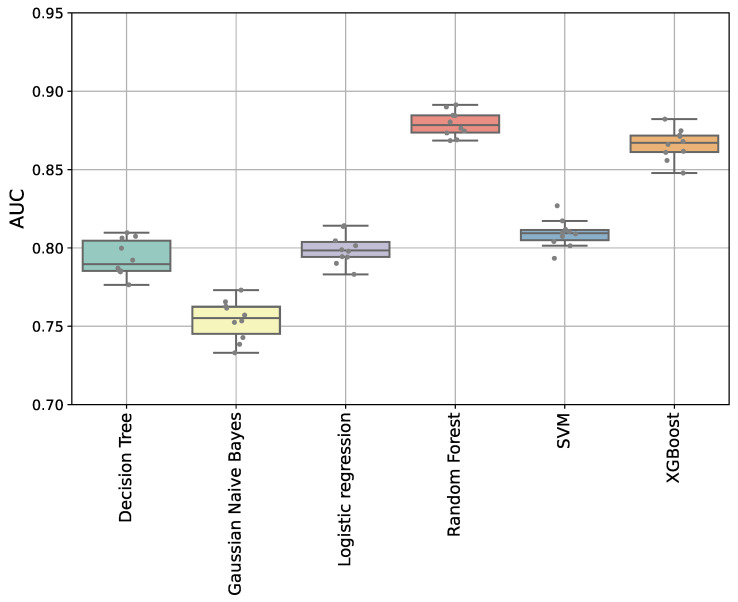
**Comparison of AUC across six machine learning algorithms.** Models evaluated were decision tree (DT), Gaussian naive Bayes (GNB), logistic regression (LR), random forest (RF), support vector machine (SVM), and XGBoost (XGB). RF achieved the highest median AUC (0.88, IQR 0.87–0.88). See Supplementary Table S2 for values.

The data were then split 80% into model training and 20% internal validation datasets, balanced for outcome, trace duration, gestational age, and fetal sex. We trained the algorithms to predict the preterm adverse outcome group using the seven FHR features and 10-fold cross validation with each fold balanced for outcome, trace duration, gestational age, and fetal sex. We then compared the performance of each predictive model using the receiver-operator area under the curve (AUC) (Figure 2, Supplementary Table S2). The random forest and XGBoost algorithms demonstrated the best performance with a mean AUC of 0.88 (95% CI 0.87–0.88) and 0.87 (95% CI 0.86–0.87, *p* < 0.001).

The relative importance of each FHR feature in the random forest model was subsequently evaluated. In a random forest model, the importance of each feature is determined by measuring how much a particular feature improves the model’s performance, averaged across all the trees within the forest. Importance varied considerably across the assessed features. Short-term variation contributed the largest proportion of the model’s predictive capacity (27.1%). This was followed by baseline heart rate and episodes of high variation contributing 16.4% and 13.8% to the model’s accuracy, respectively. Features such as accelerations and episodes of low variation also held moderate predictive value, with importances of 12.0% and 11.2%, correspondingly. Conversely, most lost beats and decelerations had diminished relative importance, contributing 5.4% and 2.4% respectively to the model’s overall predictive performance (Supplementary Figure S1).

The predictive performance of the random forest model was then evaluated for each of the individual outcomes contributing to a classification of preterm adverse outcome. The majority of outcomes exceeded an AUC of 0.80. The median AUC across all individual outcomes was 0.85 (IQR 0.81–0.89), demonstrating robust performance (Supplementary Table S3). Performance was highest for hypoxic ischemic encephalopathy (AUC 0.99, IQR 0.70–0.99, n = 7) and lowest was for a prolonged special care admission exceeding seven days (AUC 0.77, IQR 0.73–0.80, n = 161).

**Figure 3:**
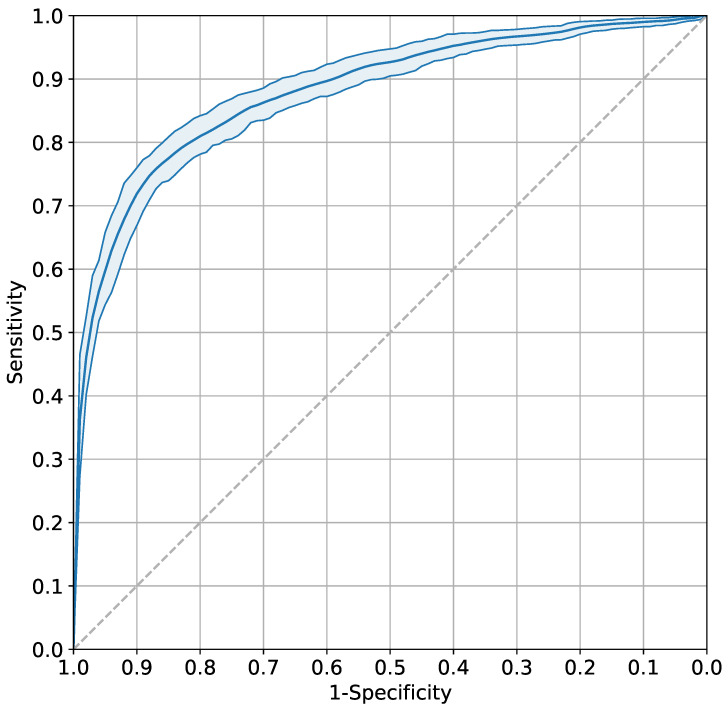
**Receiver operating characteristic (ROC) curve for the prediction of an adverse outcome in a pre-term fetus on the validation dataset.** The area under the curve (AUC) for the random forest classifier was 0.88 (95% CI 0.86–0.90), demonstrating an ’excellent’ degree of performance.

We then evaluated the model’s performance on the internal validation dataset. The model performed well with an AUC of 0.88 (95% CI 0.86–0.90, Figure 3) and demonstrated a high degree of calibration (Brier score 0.14, Supplementary Figure S2). Decision curve analysis was used to assess the net benefit of the model across a range of probability thresholds (0.01–0.99) compared to treat-all and treat-none strategies. The net benefit of the model exceeded the treat-all strategy for all probability thresholds above 0.11 (Supplementary Figure S3) and exceeded the treat-none strategy for all probability thresholds.

Three probability thresholds were evaluated for classifying pregnancies as normal or preterm adverse outcome: the Youden index, defined as the point on the receiver operating characteristic curve at which sensitivity and specificity are jointly maximised, and thresholds corresponding to 95% sensitivity and 95% specificity. Model performance at each threshold was summarised using sensitivity, specificity, F1 score, and Cohen’s Kappa (Table 3). At the Youden threshold (59.6%, 95% CI 55.6–62.9), sensitivity was 76.2% (95% CI 72.6–80.5) and specificity was 87.5% (95% CI 83.3–91.0). At the threshold selected to achieve 95% sensitivity (29.3%, 95% CI 28.9–31.6), specificity decreased to 41.4% (95% CI 33.9–49.2). Conversely, at the threshold corresponding to 95% specificity (73.6%, 95% CI 70.1–77.0), sensitivity was 59.3% (95% CI 54.0–65.6). The F1 scores at the Youden, 95% sensitivity, and 95% specificity thresholds were 81.7 (95% CI 79.6–83.9), 78.1 (95% CI 75.4–80.7), and 72.5 (95% CI 68.3–77.4), respectively. Corresponding Cohen’s Kappa values were 62.8 (95% CI 59.6–66.4), 38.1 (95% CI 30.3–46.4), and 52.3 (95% CI 46.6–58.6).

We then assessed the performance of the model for each gestational age interval (Supplementary Table S4 & Supplementary Figure S4). The median AUC exceeded 0.90 between 27^+0^ (AUC 0.93, 95% CI 0.86-0.98) and 31^+6^ weeks (AUC 0.93, 95% CI 0.89–0.97) and exceeded 0.80 for all subsequent weeks. The highest AUC observed was 0.93 (95% CI 0.89–0.97) at 31^+0^–31^+6^ weeks, while the lowest was at 36^+0^–36^+6^ weeks (0.81, 95% CI 0.77–0.85).

## 4. Discussion

We have shown machine learning algorithms can contribute substantially towards identifying high-risk pre-term pregnancies using antepartum FHR patterns. We identified a cohort of high-risk pre-term pregnancies and used a clinically validated algorithm to extract seven physiologically validated FHR features that were independent from the pitfalls of subjective assessment. We then applied machine learning algorithms to develop a high-fidelity predictive model capable of discriminating across a range of gestational ages. The model performed well when evaluated across a range of metrics on the validation dataset, including high sensitivity, specificity, F1 score, and Cohen’s kappa. Decision curve analysis also demonstrated the model significantly outperformed both treat-all and treat-none strategies.

To our knowledge, this is the first large-scale study of its kind to apply a fully automated machine learning approach to antepartum CTG in preterm pregnancies using clinically validated FHR features and objectively defined neonatal and perinatal outcomes. Unlike previous studies, which have either relied on visual interpretation of the FHR patterns or surrogate outcomes based on clinical impressions (for example, a “non-reassuring” trace), the present study is independent of subjective assessment and observer bias. This is an important distinction given the well-documented variability and limited reproducibility of human interpretation of FHR traces.

This represents an important advancement in the application of machine learning to clinical care of the pregnancy. FHR monitoring is one of the few yet affordable technologies available for immediate and real-time evaluation of fetal physiology and wellbeing, yet its clinical utility is limited by the complexity of visual interpretation. poor inter- and intra-rater reliability and high false positive rates have been linked to unnecessary intervention, including avoidable caesarean sections (and consequent preterm morbidity), as well as adverse fetal outcomes from a failure to make a timely intervention.

In current clinical practice, antepartum cardiotocography is most commonly performed in pregnancies already perceived to be at increased risk, such as those complicated by reduced fetal movements, antepartum hemorrhage, hypertensive disease, or suspected placental insufficiency. Interpretation relies on visual assessment or, in selected settings, computerised cardiotocography using rule-based criteria derived from FHR variability metrics. While randomised trials in specific high-risk phenotypes, such as growth-restricted fetuses, have demonstrated that incorporating quantitative cardiotocography measures can improve outcomes, these approaches typically apply fixed thresholds to individual features and do not exploit the full multivariate structure of the signal [52]. As a result, substantial diagnostic uncertainty persists in many common clinical scenarios, limiting the ability to distinguish fetuses that would benefit from intervention from those in whom continued surveillance is appropriate.

These results indicate machine learning algorithms possess substantial promise in outperforming clinical experts and existing systems at this task. Early detection of high-risk pre-term pregnancies is critical since progression to labor would substantially increase the risk of an adverse outcome or death. A high-fidelity system decoupled from the difficulties and inherent biases associated with visual interpretation of these signals would therefore be of significant clinical benefit.

The associations we identified between each FHR pattern and the prediction of a high-risk pre-term pregnancy are supported by previous studies. FHR accelerations are an indicator of neurological health, concomitant with a healthy response to transient umbilical cord compression and fetal movements, and suggest an absence of hypoxia [53]. Episodes of high variation are analogous to episodes of active sleep/wakefulness. Cycling between episodes of active sleep is a hallmark of normal neurological development, the absence of which has been associated with acidaemia, hypoxia, and low Apgar scores [53,54]. Low STV values have been associated with an increased risk of fetal acidaemia [55,56]. Similarly, abnormalities in basal FHRs (fetal bradycardia) are associated with an increased risk of adverse outcomes, including hypoxia, sustained umbilical cord compression, hypoxia, cardiac anomalies, and maternal hypotension [57,58].

Decelerations and their magnitude are associated with an increased risk of an adverse pregnancy outcome. Large-magnitude decelerations are known to occur in acute fetal hypoxia and acidosis [59]. Episodes of low variation are analogous to quiet/deep sleep. Prolonged episodes in the absence of high-variation episodes suggest a potential deficiency in normal neurological development [47]. Our findings show that a maximally discriminative model should incorporate all of these patterns. This is in contrast to some current clinical guidelines, which only employ a subset of features [53,60]. Frequently, one or more of these patterns are absent from a trace (e.g., accelerations or decelerations) yet do not necessarily convey an increased risk of adverse outcome [53]. In this case, a multivariate approach incorporating other such patterns is required. Historically, a failure to recognise this has resulted in unnecessary deliveries [61,62].

The predictive performance of our model mildly declined after 35^+0^ gestational weeks. These changes potentially reflect the current understanding of the physiological development of FHR patterns. Towards term, decelerations occur more frequently in normal pregnancies, subsets of which are an indicator of normal physiological responses to transient cord compression, for example [63]. As the normal neurological system of the fetus develops, cycling between quiet and active sleep also occurs more frequently, with the mean duration of an episode of low variation increasing [64,65,66]. The average short-term variability also increases with gestational age, which may diminish the distinction between normal and adverse outcome traces at later gestations [67].

Most studies developing machine learning models analyzing FHR signals have focused on the intrapartum period [36,68]. Many of these approaches rely on FHR patterns identified by human visual interpretation, limiting reproducibility and generalisability due to the well-recognised variability of expert assessment [49]. Other studies have either utilised significantly smaller datasets, did not use established clinical outcomes, or were developed using a restricted subset of pregnancies [69,70,71]. Some were designed to assign the FHR into international classifications already known to suffer from poor performance. Some studies have analyzed only short segments of the FHR trace, such as the final 30 min prior to delivery [36]. This substantially restricts generalisability and introduces bias, as the length of a trace prior to acquisition is frequently unknowable and longer traces are generally performed in response to concerning features observed during monitoring.

Several potential sources of bias should be considered when interpreting these findings. The dataset reflects antepartum monitoring performed in a tertiary-care setting, where cardiotocography is undertaken for specific clinical indications rather than as a universal screening test. As a result, pregnancies classified as having a normal outcome in this study are not representative of an unselected low-risk population but instead reflect cases in which monitoring was clinically indicated and no adverse outcome subsequently occurred. This potentially introduces referral and indication bias, particularly when comparing normal and preterm adverse outcome cohorts. We have sought to mitigate this through the thorough application of inclusion and exclusion criteria for assignment to the NPO cohort. The relative enrichment of adverse outcomes compared with uncomplicated controls, while necessary to enable robust model development, does not reflect population-level prevalence. To mitigate these effects, we employed propensity score matching, balanced training and validation splits, and evaluated model calibration and clinical utility in addition to discrimination. The study also spans multiple decades of clinical practice, during which obstetric management strategies and indications for fetal monitoring have evolved. Importantly, this study was not designed to define or evaluate a screening pathway for antepartum cardiotocography. Rather, it aims to quantify the extent to which clinically validated FHR features contain discriminative information for adverse outcomes in preterm pregnancies. The findings therefore provide a foundation for future work to explore specific clinical applications, such as supporting early recognition of fetal compromise within existing surveillance pathways or reducing unnecessary intervention through improved risk stratification. Further details regarding dataset composition, governance, and known sources of bias are described in the OxMat dataset resource [31], which provides a comprehensive account of the underlying data used in this study.

The generalisability of this model to external datasets and different healthcare settings warrants careful consideration. Factors supporting transferability include the use of clinically validated FHR features that are routinely available across monitoring platforms and a modeling approach that does not depend on site-specific metadata, high-resolution waveform data, or proprietary device outputs. These characteristics may facilitate adaptation to settings where computational resources and data infrastructure are limited. However, differences in population risk profiles, referral patterns, monitoring indications, gestational age distributions, and signal quality may affect performance when applied to external cohorts. As such, prospective external validation in independent populations, particularly in low-resource settings, is an essential next step before clinical implementation.

In the decision curve analysis, probability thresholds were interpreted in the context of typical antenatal management decisions for pre-term pregnancies rather than as fixed treatment cut-offs. Lower thresholds reflect clinical scenarios in which the perceived risk of adverse outcome may prompt increased surveillance, hospital admission, or repeat cardiotocography, whereas higher thresholds correspond to decisions involving more intensive intervention, such as administration of antenatal corticosteroids, magnesium sulfate for neuroprotection, or planning for early delivery. The model demonstrated net benefit across a broad range of thresholds, indicating potential utility in supporting risk-informed decision-making at multiple stages of care. Importantly, these thresholds are not intended to prescribe automated actions but to provide probabilistic information to assist clinicians in weighing risks and benefits in conjunction with existing guidelines and clinical judgement.

A direct comparison of our results with existing techniques is limited by the current absence of comparable studies in this clinical domain. To our knowledge, there are no published machine learning approaches that analyze antepartum cardiotocography in pre-term pregnancies using clinically validated FHR features and objectively defined neonatal or perinatal outcomes. Existing computational studies predominantly focus on intrapartum recordings (where the fetal environment is different), term pregnancies, or surrogate outcomes such as expert CTG classification, which differ substantially in both physiological context and clinical intent. The lack of established antepartum pre-term comparators therefore reflects a gap in the literature rather than an omission in comparative evaluation and underscores the novelty of the present study.

The FHR features used in this study were selected *a priori* based on the Dawes–Redman method of computerised cardiotocography, a widely used and clinically validated framework for antepartum fetal surveillance [8,47]. The Dawes–Redman system was originally developed to assess whether an antepartum cardiotocography trace meets predefined criteria for normality, primarily at term, with the aim of identifying fetuses at low risk of hypoxia or acidaemia and safely reducing unnecessary intervention. In routine clinical practice, it is therefore most often applied as a rule-based tool to confirm normality rather than to quantify degrees of abnormal risk, and its outputs are binary in nature, indicating whether criteria are met within a given recording period. While Dawes–Redman criteria have proven value for excluding fetal compromise, particularly in term pregnancies, they are not designed to provide probabilistic risk stratification or to discriminate across heterogeneous adverse outcomes, especially in the pre-term setting. Pre-term FHR patterns differ substantially from those observed at term due to physiological immaturity of the autonomic and central nervous systems, and strict application of normality thresholds may therefore be inappropriate or overly conservative. In addition, the Dawes–Redman framework does not integrate information across features in a multivariate manner, nor does it adapt to gestational age or outcome-specific risk profiles to the extent demonstrated in this study. In contrast, our approach uses the same physiologically grounded Dawes–Redman features as continuous quantitative inputs to a machine learning model, rather than as fixed thresholds defining normality. This allows the model to learn complex relationships between complementary aspects of FHR behavior and adverse outcomes across a range of pre-term gestations. By retaining clinically interpretable features while abandoning rigid rule-based decision criteria, this strategy enables more nuanced risk estimation, improved discrimination, and calibration while preserving transparency and applicability across monitoring systems. We therefore view this work not as an alternative implementation of Dawes–Redman criteria but as an extension that leverages their physiological validity within a data-driven framework better suited to pre-term risk stratification.

Preterm adverse outcomes are inherently multifactorial, reflecting the interaction of fetal physiology with maternal demographic, clinical, and obstetric factors. In this study, we intentionally restricted the model inputs to fetal heart rate-derived features to isolate the predictive contribution of antepartum cardiotocography and to maintain interpretability and portability across clinical settings. Maternal characteristics and clinical variables were therefore not incorporated into the current models. Integration of multimodal information, including maternal demographics, medical comorbidities, pregnancy complications, and laboratory or ultrasound findings, represents an important avenue for future work and may further enhance predictive performance and clinical utility.

From an implementation perspective, this approach could be integrated into clinical workflows in several ways. One potential pathway is deployment as a stand-alone decision support tool that processes antepartum cardiotocography data in real time and returns an interpretable risk estimate to clinicians during routine surveillance. Alternatively, the model could be embedded within electronic health record systems, enabling automated analysis of stored cardiotocography traces and longitudinal risk tracking alongside other clinical information. In both scenarios, the output is intended to complement existing antenatal care pathways by providing probabilistic risk stratification rather than prescriptive recommendations, thereby supporting clinician judgement and shared decision-making. Prospective evaluation of usability, workflow integration, and clinical impact will be essential steps prior to deployment. More broadly, the integration of quantitative decision-support tools into clinical workflows has been explored across other domains of healthcare delivery, including hospital-level performance and service evaluation, highlighting the growing role of data-driven methods in supporting complex clinical and organisational decision-making [72]. Within this context, antenatal risk stratification represents a particularly suitable application, given the need to balance timely intervention against the risks of unnecessary treatment.

While deep neural network architectures are a clear consideration for analyzing such data, there are several important advantages to this current approach [73,74,75,76]. The FHR patterns in our model are physiologically driven and their contributions to the model are easily interpretable, enabling simplified interrogation of results and the potential to advance our understanding of these patterns. These results will also serve as an important first benchmark for future studies, where more complex modeling approaches could be evaluated. A simple, understandable algorithm relying on low-cost and accessible technology is more readily deployable across diverse healthcare settings. FHR monitoring is one of only a few technologies available offering real-time appraisal of fetal physiology. Alternative approaches are either costly, time-consuming, require extensive training, or are inaccessible, particularly in low-resource settings. Even where cardiotocography is available, the expertise required for reliable interpretation remains a major barrier. In these contexts, robust and effective machine learning algorithms may play a critical role in unlocking the full potential for FHR monitoring.

The present findings should therefore be viewed as establishing the latent diagnostic signal available within antepartum FHR data, rather than defining a single clinical application. Notably, the observed effect sizes were achieved across a heterogeneous cohort of preterm adverse outcomes, without conditioning on specific clinical indications or phenotypic subgroups. It is plausible that model performance and clinical utility would be further enhanced when applied to more narrowly defined contexts, such as pregnancies monitored for suspected placental dysfunction, recurrent reduced fetal movements, or other high-risk presentations. This work, therefore, lays the foundation for future studies to determine in which specific clinical scenarios the additional predictive resolution afforded by machine learning analysis of cardiotocography can meaningfully reduce both stillbirth and unnecessary iatrogenic preterm birth.

## 5. Conclusions

Electronic FHR monitoring remains an important and irreplaceable investigation in the assessment of fetal wellbeing, yet its clinical utility is limited by the challenges of reliable interpretation, often resulting in avoidable adverse outcomes. In this study, we demonstrate that that machine learning based on analysis of antepartum FHR patterns can accurately detecting high-risk preterm pregnancies. This bespoke model may enable earlier and more accurate diagnosis and facilitate better management of these pregnancies. Prospective external validation will be essential to determine its impact on clinical outcomes.

## Figures and Tables

**Table 1:** **Study population characteristics.** Summary of maternal and fetal characteristics for normal pregnancy outcome and preterm adverse outcome cohorts. Values are shown as median (interquartile range) or counts, with effect sizes reported where applicable.

Feature	Normal Outcome	Preterm Adverse Outcome	*p*	Effect Size
FHR traces	4014	4867	–	–
Maternal age	29 (25–33)	31 (26–35)	<0.01	Small
Viable parity	1 (0–1)	0 (0–1)	<0.01	Small
Non-viable parity	0 (0–1)	0 (0–1)	<0.01	Small
BMI	23.5 (21.2–25.9)	24.9 (22.0–29.4)	<0.01	Medium
Fetal sex	Male: 1960	Male: 2375	0.99	–
	Female: 2054	Female: 2492		

**Table 2:** **FHR features by outcome group.** Median (interquartile range) values for each extracted feature in normal and preterm adverse outcome pregnancies, with corresponding effect sizes.

Feature	Normal Outcome	Preterm Adverse Outcome	*p*	Effect Size
Accelerations	5 (3–8)	3 (1–5)	<0.001	−0.4 (Large)
Baseline heart rate	138 (132–144)	139 (132–145)	<0.001	0.0 (Small)
Decelerations	0 (0–1)	0 (0–1)	<0.001	0.1 (Small)
High variation (minutes)	7 (3–14)	2 (0–7)	<0.001	−0.4 (Large)
Low variation (minutes)	0 (0–1)	4 (0–22)	<0.001	0.4 (Large)
Most lost beats	8 (6–11)	11 (8–18)	<0.001	0.2 (Medium)
Short-term variation	9 (8–11)	7 (5–9)	<0.001	−0.5 (Large)

**Table 3:** Evaluation of the random forest model using the validation dataset. The performance of the predictive model was evaluated using three different probability thresholds. Cases above the threshold were classified as preterm adverse outcome pregnancies; those below were designated as normal. The Youden threshold is the probability threshold at which the sensitivity and specificity are maximal. Sensitivity (true positive rate) measures the proportion of actual preterm adverse pregnancy outcomes that are correctly identified by the model. Specificity (true negative rate) quantifies the proportion of actual normal outcome pregnancies accurately classified by the model. The F1 score is the harmonic mean of precision (the proportion of true positives among the identified positives) and sensitivity, providing a balanced measure of a model’s performance. Cohen’s Kappa measures the degree to which the classifications made by the algorithm agree with the true classifications, accounting for the agreement that would be expected by random chance. Values in brackets denote the 95% confidence intervals.

	Threshold(%)	Sensitivity(%)	Specificity(%)	F1 Score(%)	Cohen’s Kappa
Youden	59.6	76.2	87.5	81.7	62.8
	(55.6–62.9)	(72.6–80.5)	(83.3–91.0)	(79.6–83.9)	(59.6–66.4)
95% Sensitivity	29.3	–	41.4	78.1	38.1
	(28.9–31.6)		(33.9–49.2)	(75.4–80.7)	(30.3–46.4)
95% Specificity	73.6	59.3	–	72.5	52.3
	(70.1–77.0)	(54.0–65.6)		(68.3–77.4)	(46.6–58.6)

## Data Availability

The data used in this study comprise sensitive patient-level clinical information and are subject to ethical and legal restrictions. As such, they cannot be made publicly available. Access to the data may be considered on reasonable request to the corresponding author, subject to approval by the relevant institutional review boards and data governance bodies. The code used for data processing, feature extraction, and model development is proprietary and is being developed for future commercial use. Consequently, it cannot be shared publicly at this time.

## Supplementary Materials

The following supporting information can be downloaded at: https://www.mdpi.com/article/10.3390/bioengineering13020203/s1.
